# Relevance of Students’ Goals for Learning Engagement and Knowledge Gains in an Online Learning Course

**DOI:** 10.3390/bs13020161

**Published:** 2023-02-12

**Authors:** Martin Daumiller, Raven Rinas, Markus Dresel

**Affiliations:** Department of Psychology, University of Augsburg, 86159 Augsburg, Germany

**Keywords:** online learning, achievement goal, motivation, goal orientation

## Abstract

Online courses are an important form of educational delivery worldwide, yet students differ in how well they learn from them. Following psychological and educational research, students’ goals can be considered relevant personal predictors of these differences. In the present study, we strive to better understand differences in students’ learning engagement and learning gains and investigate how they are related to their achievement goals. We distinguish between two types of mastery goals (task and learning goals) along with performance approach goals and performance avoidance goals. We constructed an online course and assessed 182 undergraduates’ goals and prior knowledge before, as well as their knowledge gains after learning with the course. Through learning analytics, we measured learning engagement during the course based on nine objective indicators concerning usage, time, and clicks. Structural equation modelling showed that task goals but not learning goals were beneficial for learning engagement and, in turn, learning gains. This paints a more nuanced picture of how mastery goals matter and illuminates how students’ goals form a relevant premise for successful online learning. While online courses may differ in design and support provided, our findings imply that personal learner characteristics, such as student motivations, should also be acknowledged.

## 1. Introduction

Online courses are a central element of educational systems and are becoming increasingly important. Throughout the past years, learning with online courses has rapidly increased and has been strongly incorporated into primary, secondary, and tertiary education [1,2]. For example, in the USA during the academic year 2019–2020, the percentage of students enrolled in distance education courses at postsecondary institutions was 51.8% [3]. Adding to this, online learning is considered one of the most important forms of educational delivery in the world [4], and the recent shift to online teaching and learning during the COVID-19 pandemic provided a glimpse of what more is to come. However, this increasing trend of learning in online courses also comes with certain challenges, such as lower participation and completion rates [4,5], making knowledge about how students differ in their engagement and learning within online courses crucial (see also [6]).

Despite the rising use and relevance of online learning, research examining factors that drive students to successfully learn in such formats is still in its early stages. At the same time, knowledge about learning in traditional face-to-face classroom settings cannot be readily transferred to online learning contexts, as learning in classrooms and online courses systematically diverge. Aside from format differences in terms of modes of interaction, accessible information, and (non-)linearity of design [7], online learning is characterized by more autonomy and a strong need for self-regulation in learning [8,9]. While the design and features of online courses, such as social support and ease of use, are central to learning, characteristics of the learners themselves are also important for successful online learning [10]. Within a given online course, there can be substantial differences in the extent to which students engage. Consequently, to learn successfully in online courses, high levels of motivation and continuous learning engagement are required and need to be fostered [11].

In the present research, we strive to better understand differences in students’ engagement in online courses and investigate how they are related to their motivations, as well as how they matter for learning performance. To this end, building on previous psychological and educational research, we conceptualize motivation using an achievement goal approach. Combining traditional data collection methods and learning analytics, we show that mastery goals are particularly beneficial for engagement and, in turn, learning gains.

## 2. Achievement Goals and Learning in Online Courses

Achievement motivation pertains to the energization and direction of competence-relevant behavior and describes why and how people strive toward competence and away from incompetence. As such, achievement motivation is essential for how we experience and behave in achievement contexts, including learning in online courses. Moreover, motivation is dynamic and composed of personal as well as contextual aspects. Thus, for learning in online courses, it is important to consider the specific motivations that students have with reference to an upcoming course as opposed to their general motivational tendencies. While the former motivations do not (necessarily) reflect why individuals decide to participate in a course in the first place, they shed light on differences in how students are motivated regarding this context. A prominent approach in educational psychology to describe such motivations is the achievement goal approach [12].

Achievement goals represent the “different ways of approaching, engaging in, and responding to achievement situations” [13]. They form cognitive representations of competence-related end states in achievement contexts that an individual is committed to approaching or avoiding [12]. Different types of achievement goals with which students can approach a given online course with can be distinguished [14]. Given that these goals represent what individuals want to reach when participating in a course, they act as a motivational basis for the interpretation of learning situations and self-regulation therein. Thus, close links with learning engagement and learning outcomes are to be expected. Indeed, previous research has shown that achievement goals are associated with self-regulated learning [15,16], learning engagement [17,18], and academic achievement [19].

Following the achievement goal approach, different types of goals have been suggested to matter in achievement contexts. Although further distinctions have been discussed (for a contemporary overview see [20]), on a minimal level, three fundamental types of goals should be distinguished: mastery goals, performance approach goals, and performance avoidance goals. Mastery goals are characterized by a focus on task mastery and personal improvement. Based on their orientations, they can be further differentiated (see e.g., [21,22,23,24]), most notably depending on whether an individual is oriented at conducting tasks right (here termed task goals), or at the improvement of their own competencies (learning goals). Given these foci, mastery goals can be considered highly functional in learning contexts when it comes to engagement and learning gains: they are directed at increasing levels of competence by acquiring the knowledge or skills developed through engaging in a learning task [25,26,27]. It is worth noting that prior research typically assumes that mastery goals matter for learning outcomes through differences in students’ engagement; however, empirically, this proposed mediation has seldom been tested [28].

Aside from mastery goals, performance approach goals constitute striving to be better than others, and performance avoidance goals are focused on avoiding doing worse than others. Similar to mastery goals, performance goals can be further distinguished based on whether they are directed at normative comparisons regarding performance (normative goals), or at competence demonstration (appearance goals). For theoretical and conceptual clarity, we focus performance goals in the present work exclusively on normative comparisons [22,29]. Performance approach goals combine a favorable approach orientation with a focus on performance that facilitates performance efforts channeled toward normative standards, frequently eventuating in high levels of performance [25]. However, this might also distract students from deep learning, as reflected in a meta-analysis by Payne, Youngcourt, and Beaubien [26], in which no significant associations with learning gains were found despite increased (adaptive as well as maladaptive) learning processes [30,31]. In contrast, performance avoidance goals combine two negative aspects (focus on performance and avoidance valence), rendering clearly negative effects. Such goals are linked to increased anxiety, task distraction, and helpless engagement patterns [32]. They have also been associated with reduced learning gains [26] and more maladaptive learning engagement of students in the form of more procrastination, surface processing, and disorganization, as well as less deep processing [27,33,34,35].

As previously described, these general mechanisms of achievement goals may not readily transfer to online learning contexts (see [14]). Regarding online learning, only a few studies so far have investigated the relevance of achievement goals. However, accumulating evidence suggests that achievement goals, and in particular mastery goals, matter for online learning. For example, Xie and Huang [36] investigated 132 students in a collaborative college-level online course and found that students pursuing strong mastery goals exhibited more frequent participation in online learning activities and reported substantial perceived learning gains. Yeh et al. [37] found in a sample of 93 undergraduate and graduate students participating in various online courses that only mastery goals, but not performance goals, were positively related to supportive online learning behaviors and, by extension, expected grades mediated by increased use of self-regulated learning strategies. Furthermore, de Barba, Kennedy, and Ainley [14] found that for students participating in a MOOC (Massive Open Online Course), mastery approach goals, assessed retrospectively after the course, were associated with students’ quiz attempts and, in turn, their final grades. In their study, de Barba, Kennedy, and Ainley [14] considered quiz attempts as one aspect of engagement, in addition to video hits.

When interpreting the current state of this line of research, three points are especially in need of attention. First, beyond research in the online learning context, mastery goals have typically been considered on a superordinate level in that they are not further distinguished into task and learning goals. Studies across different populations suggest different patterns of learning processes depending on whether individuals are focused on task or learning standards [22,38,39,40,41,42]. However, it is not yet clear how task goals and learning goals might specifically operate for such learning processes. As learning goals are focused on improving individuals’ own competencies, they might be superior for learning processes compared to goals focused on doing tasks right, as they may connect learning closer to one’s self-worth. Conversely, task goals might be superior to learning goals in learning settings, as learning goals might distract learners from covering the full breadth of content to be learned (e.g., due to finding certain aspects particularly interesting and placing a focus on them), which might especially be the case in highly autonomous learning settings, such as online courses. As the research and practical implications drawn from these findings can differ substantially, further investigations into the different facets of mastery goals are necessary. 

Adding to this, performance goals remain little understood in online learning contexts, where studies have either omitted them altogether [14], or have not considered them relevant due to a lack of direct contact with peers in remote learning contexts [43]. Therefore, we also see a need for more research aimed at investigating the significance of performance goals, especially as pressure and concerns about other students may still be prevalent regardless of their physical presence. Thus, aside from investigating mastery goals in more detail, performance approach and performance avoidance goals should also be studied for a comprehensive understanding of how achievement goals matter for learning in online courses. 

Lastly, in motivation research in general, self-reports are typically used to assess engagement, despite this construct also encompassing behavioral elements that hold the possibility of being assessed from a more objective perspective. The use of self-reports in turn can lead to a host of problems associated with same-source bias, desirable answering, and understandability [44], which need to be overcome by assessing engagement through other means. In the present work, we therefore consider learning engagement using a broad array of learning analytics indicators concerning how exactly students interacted with the online course.

## 3. Learning Engagement and Learning Gains in Online Courses 

Learning engagement can be defined as the time and energy students invest in educationally purposeful activities [45]. Research has consistently found learning engagement to be a key contributor to success in educational contexts. Importantly, learners who adopt a more engaged approach to learning are more likely to have increased learning gains [17]. However, there is also variability in students’ learning engagement, making it necessary to understand what individual factors might lead to different levels of engagement. As previously described, following a motivation perspective, achievement goals constitute one such relevant factor.

For examining learning engagement and learning outcomes in online contexts, a unique opportunity to capture a more reliable understanding of these interrelations becomes apparent. Learning analytics paired with resulting log data from online courses represent a promising opportunity to measure and analyze how students engage with the different course materials in terms of specific interactions (e.g., views, clicks, posts, scrolls). Such objective indicators of engagement mark an important development within research on student learning, as they have the potential to overcome limitations from self-report measures and are minimally disruptive.

Indeed, several studies have found objective measures of engagement within online courses to act as meaningful indicators of students’ learning involvement and persistence [14,46,47,48,49]. These objective forms of engagement have also been linked to different learning outcomes in theoretically sensible ways. For example, Bonafini, Chae, Park, and Jablokow [46] found that students who participated in a MOOC had a higher probability of course achievement when they completed more forum posts and watched more videos. Moreover, as previously noted, de Barba, Kennedy, and Ainley [14] defined students’ engagement as their video hits and quiz attempts, which were positively linked to their interest and final grades. Adding to this, Xiong, Li, Kornhaber, Suen, Pursel, and Goins [49] assessed students’ learning engagement as a latent variable through the indicators of videos watched, forum posts, and number of assignments and quizzes completed, which was found to be positively predicted by motivation and resulted in higher student retention. To this end, construing engagement as a latent variable may be particularly advantageous to reflect the overarching psychological construct of engagement as opposed to only focusing on single indicators.

In terms of differentiating which objective indicators of learning engagement within online courses are more relevant than others, a consensus within the literature has yet to be reached [50]. Nevertheless, important criteria can be drawn from prior studies [14,46,49]. To provide more comprehensive and reliable insights, several objective indicators of learning engagement should be simultaneously examined. Moreover, certain indicators—particularly page views, video views, and interactions with quizzes and assignments—have consistently been found to be meaningfully and significantly related to students’ learning outcomes. These specific indicators are also commonly reported within studies investigating objective learning engagement in online courses and can therefore be more readily compared with existing research. Based on these considerations, in the present study, we used the objective engagement indicators of page views (regarding core lessons, additional information, videos, and quizzes), clicks (within core lessons, additional information, assessments, and quizzes), and the total time spent on the course.

## 4. The Present Research

As online learning continues to expand in higher education, the effects of pursuing different achievement goals on students’ engagement in online courses and the resulting learning gains need to be analyzed in more depth. In the present research, we follow up on this research gap. Aside from considering achievement goals from a more differentiated perspective by distinguishing task and learning facets of mastery goals, we operationalize learning engagement based on multiple objective indicators concerning how exactly students interacted with the online course. Through this, we seek to provide an alternative to the common use of self-reports for assessing engagement. 

To test the relevance of achievement goals for learning engagement in online learning courses and how this consequently matters for learning gains, we put forth the following hypotheses based on the theoretical and empirical points previously discussed:

- Hypothesis 1: Task and learning goals are positively associated with learning engagement.
- Hypothesis 2: Performance avoidance goals are negatively associated with learning engagement.
- Hypothesis 3: Learning engagement is positively associated with learning gains.

Given the split nature of performance approach goals and the mixed findings reported for this type of goal, we did not formulate a directed hypothesis for them. Furthermore, we presumed that both task and learning goals would result in positive effects but tested for potential differences between these two facets of mastery goals on an explorative level.

## 5. Method

To answer our research questions, we constructed an online course on psychological research methods embedded in the curriculum of a psychology lecture and invited 182 students to participate in it over two weeks in November 2019. The study was conducted in full accordance with the Ethical Guidelines of the German Association of Psychologists and the American Psychological Association. The full anonymity of all participants was assured. We had no reason to assume that completing our survey would have any negative effects on the participants. We provide the online course used as an open educational resource and include all data and code underlying this research in an open repository (https://osf.io/gp6h3/).

### 5.1. Participants and Procedure

The participants of this study were students attending an introductory lecture on psychology. Typical for this population, the participants were mostly women (135 identified as women, 45 as men, 2 as diverse), had an average age of 21.4 (*SD* = 2.6) years, and were in their first year of university. The students had not yet dealt with the topic of the online course (research methods) within their curriculum.

The online course contained 3 core lessons (including 10, 3, and 9 pages of learning content, respectively), 3 videos, 7 pages of additional helpful information, 16 quizzes, and 3 end-of-lesson assessments. It was constructed based on an earlier online course developed by Daumiller and Dresel [51]. We piloted the adapted course regarding understandability, relevance of content, potential technological problems, and ease of use with five students. The average time spent on the online course was 122 minutes (*SD* = 70). When accessing it for the first time, the participants were asked to complete a survey to assess their achievement goals and baseline knowledge. During the online course, we measured their learning engagement in the form of log data corresponding to the indicators of views, clicks, and total time. Directly after completing the online course, the students were required to participate in another knowledge test covering the topics within the course to gauge their learning gains.

### 5.2. Measures

We used the scale by Daumiller, Dickhäuser, and Dresel [20] to measure task approach goals (3 items; e.g., “… my goal is to fulfill the different requirements very well”; ω = 0.90), learning approach goals (4 items; e.g., “… my goal is to expand my knowledge as much as possible.”; ω = 0.93), performance approach goals (4 items; e.g., “… my goal is to be better than the other students.”; ω = 0.94), and performance avoidance goals (4 items; e.g., “… my goal is not to be worse than the other students”; ω = 0.94). A confirmatory factor analysis (CFA) confirmed the four-dimensional structure (CFI = 0.96, TLI = 0.96, SRMR = 0.05). All items were directed at the online course (item stem: “In this online course…”) and answered on a 5-point Likert-type scale ranging from 1 (do not agree at all) to 5 (agree completely).

As indicators of students’ learning engagement during the course, we used log data concerning (a) the total amount of time students spent on the course, (b) how many times they viewed core lesson pages, (c) how many videos they viewed, (d) how many times they viewed additional information pages (containing supplemental information such as tips for reading research articles and a dictionary with keywords), (e) how many times they viewed quizzes, (f) how active they were within the core lesson pages, (g) how active they were within the additional information pages, (h) how many answers they submitted within the assessments, and (i) how many answers they submitted within the quizzes. We modeled residual correlations between closely corresponding and partly dependent indicators (e.g., lesson views with lesson clicks). A CFA confirmed the presumed one-dimensional structure and supported modeling learning engagement as a latent factor (CFI = 0.97, TLI = 0.95, SRMR = 0.05).

We measured students’ content knowledge concerning the online course by administering a slightly adapted and expanded multiple-choice test by Daumiller and Dresel [51] containing 4 questions before the start of the course as well as the same questions along with 6 further, more difficult questions after the students finished the online course. Each question contained four multiple-choice answers. Based on the proportion of correct items (pre-test: *M* = 0.64, *SD* = 0.13, Min = 0.19, Max = 0.88; post-test: *M* = 0.65, *SD* = 0.11, Min = 0.38, Max = 0.90), we subsequently computed the residual change scores between these two knowledge test scores to describe students’ learning gains during the course. Given that the post-test was more difficult than the pre-test, it should be noted that the change scores do not reflect the absolute amount of learning gains for each student (which we were not interested in), but instead allow us to quantify differences between the participating students with regard to their learning gains. 

### 5.3. Analyses

We estimated a structural equation model in which learning gains were regressed on learning engagement (as a latent variable based on nine indicators) that was in turn regressed on the four achievement goals. We allowed for direct effects from goals on learning gains. We estimated the direct effects as standardized partial regression coefficients and obtained indirect effects by combining the specified coefficients for direct effects; their statistical significance was tested with z-tests. The model was estimated in R version 4.1.1 (R Core Team, Vienna, Austria, 2021) using the lavaan package version 0.6–9 and MLR as an estimator. There was no missing data.

## 6. Results

We present descriptive statistics as well as bivariate correlations between all variables in Table 1. In line with prior research on achievement goals, we found rather high levels of task and learning goals and lower levels of performance goals. All goals contained a substantial amount of intra-individual variability (as reflected in their standard deviations), indicating that the different students started the course with different compositions of goals.

**Figure 1:**
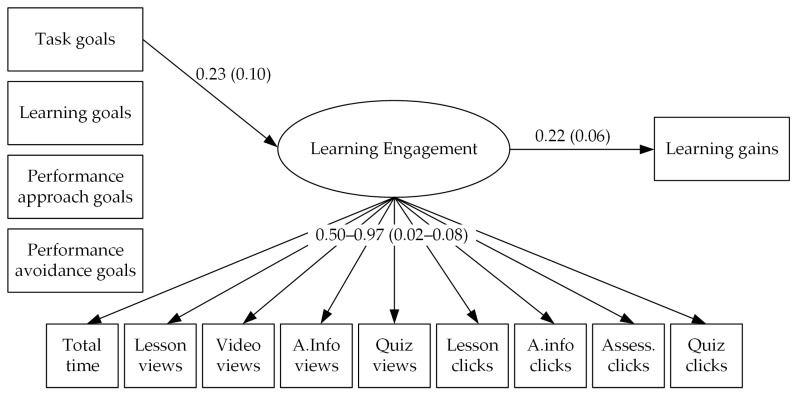
Results of analyzing the associations between achievement goals, learning engagement, and learning gains (only statistically significant effects are visualized, residuals and their correlations are not presented). We present standardized regression weights/factor loadings with their standard errors in brackets.

The results of the structural equation model (CFI = 0.96, TLI = 0.95, SRMR = 0.05) are visualized in Figure 1 and indicate that task approach goals had a positive, statistically significant effect on learning engagement, while no significant effects were observed for learning approach goals or the two performance goals (learning goals: –β = 0.17, *SE* = 0.10; performance approach goals: –β = 0.14, *SE* = 0.10; performance avoidance goals: –β = 0.07, *SE* = 0.12). In fact, the two regression weights of learning engagement on learning goals and task goals statistically significantly differed from each other (reflected in a clear deterioration of the model fit when restricting them to be equal: Δχ^2^ = 4.57, *df* = 1, *p* = 0.03). This means that students who focused on doing their tasks right exhibited more learning engagement than students with weaker task goals, and in particular also more engagement than students with strong learning goals. 

Learning engagement in turn had a positive, statistically significant effect on learning gains. This means that students who had higher learning engagement based on our log data indicators also improved their knowledge from pre- to post-test more than those with less learning engagement. According to Acock [52], both effects can be considered moderate. Furthermore, there was a small, statistically significant indirect effect from task goals via engagement on learning gains (β = 0.05, *SE* = 0.02), indicating that task goals are positively associated with learning gains through students putting forth increased learning engagement.

## 7. Discussion

As online courses are an important form of educational delivery worldwide, yet students vary substantially in how well they learn from them, we aimed to investigate differences in students’ learning engagement and how they are related to students’ motivations and learning gains. Following an achievement goal approach, we distinguished between two mastery goals, namely task and learning goals, and included performance approach and performance avoidance goals. Besides this detailed view of students’ achievement goals, we expanded on prior motivation research that primarily relied on self-reports by combining traditional data collection methods and novel learning analytics techniques and measuring learning engagement based on a broad variety of objective indicators. Our finding that task goals are especially beneficial for learning engagement, and in turn, learning gains, paints a more nuanced picture of how mastery goals matter and illuminates how students’ goals form a relevant premise for successful online learning.

Confirming our expectations and in line with prior findings [14,46], we found that learning engagement measured via objective indicators within the online course did indeed matter for students’ learning gains. It should be borne in mind that our operationalization primarily focused on behavioral aspects of engagement, however, an increased relevance of engagement might have been found had we also expanded our conceptualization of learning engagement to include cognitive and affective aspects more strongly. This finding highlights the importance of designing online courses in ways that spur engagement, as well as considering the role of personal learner characteristics that might impact learning engagement. Regarding the latter, our results indicate that students’ motivations expressed in the form of achievement goals constitute personal factors that impact their engagement in online courses.

In terms of the linkages with learning engagement, our findings suggest that across different types of learning environments, mastery approach goals are positively related to learning outcomes (see also [53]). The discovery that task goals particularly matter (when compared to learning goals) for learning engagement and learning gains requires a closer look into the relevance of mastery goals. Our findings imply that task and learning goals might indeed differ in how they matter for students’ learning [22,38,39,40,41,42], and provide additional evidence that learning goals may not be facilitative in all contexts [54]. At least within clearly defined learning contexts, such as our online course, it might be the case that task goals are superior to learning goals in terms of the learning processes that they instill, as learning goals could distract learners from covering the full breadth of content to be learned. This might be due to students with strong learning goals finding certain aspects of the learning content interesting and focusing primarily on them. The descriptively negative regression weight that we observed for learning goals provides additional indications to this end. Such effects of learning goals might be especially likely in highly autonomous settings (such as learning in online courses). In our study, we designed the knowledge tests to operationalize learning gains by content-validly reflecting the full range of content within the course. Thus, it makes sense that learning goals may have served to foster deeper specialized knowledge gains as opposed to an understanding of all relevant content to be learned (what our measures touched on). We recommend future research to follow up on this by including other means, such as interviews, to inquire about students’ learning processes in more detail. 

Another important takeaway is that although we also examined performance goals—which have only scarcely been considered in past research examining the relevance of goals for online learning (see [14,43])—no statistically significant effects were identified for these goals with students’ learning. While this might not be surprising for performance approach goals (which we did not have directed expectations for and are often not strongly, and with substantial heterogeneity, linked to academic performance [55]), this stands in contrast to the negative associations that we hypothesized for performance avoidance goals that are consistently documented in the literature regarding academic experiences and behaviors, e.g., [23,56]. However, it should be borne in mind that this might to a certain extent have also been a function of the features of the online course, which may not have provided enough opportunities for social comparisons (e.g., by comparing one’s quiz/assessment scores with those of other students). Therefore, future research should consider a broad array of course features and how these might matter for the effects of performance goals. Specifically, it might be the case that performance goals matter less for achievement behaviors within settings in which social comparisons are restricted or fellow students are not directly present [57,58]. 

When interpreting our findings, three key limitations should be considered. First, our sample was based on a single online course with unique features, limiting the generalizability and comparability of our findings. This could be improved in future research by extending the present study to courses with different designs. Second, we focused on engagement from an objective and behavioral perspective; however, additionally assessing emotional and cognitive facets of engagement would be an interesting and important direction for future research. Third, we focused on learning gains as an outcome—an interesting perspective for future research would be to follow up on differences in students’ learning engagement based on differences in their prior knowledge, which in turn might interact with students’ goals in a complex manner (e.g., learning goals may be more relevant drivers for students with little prior knowledge). Nevertheless, our findings already point to first practical implications. Specifically, task goals should be supported to allow for more successful online learning experiences. This may be facilitated through (1) directly influencing these goals by stressing the importance of task mastery and putting the respective goals into writing, and (2) supporting these goal-striving processes by an arrangement of contextual features emphasized in the online course [59].

In conclusion, our findings contribute to a better understanding of how established models of educational research can be applied to shed light on students’ engagement and learning gains in the increasingly important context of online learning. While online courses may differ in design and support provided, personal learner characteristics, such as individual motivations, should also be acknowledged.

## Figures and Tables

**Table 1:** Descriptive Statistics and Bivariate Correlations.

	*M*	*SD*	1	2	3	4	5	6	7	8	9	10	11	12	13
Achievement goals															
[1] Task approach goals	3.92	0.83													
[2] Learning approach goals	4.08	0.80	0.69												
[3] Performance approach goals	2.15	1.00	0.05	−0.11											
[4] Performance avoidance goals	2.59	1.06	0.15	0.04	0.74										
Learning engagement															
[5] Total time	122	70.3	0.11	0.04	−0.02	0.07									
[6] Lesson views	42.7	23.6	0.08	−0.01	−0.07	0.02	0.54								
[7] Video views	1.75	2.27	0.20	0.07	0.03	0.05	0.45	0.48							
[8] Additional information views	9.27	8.74	0.22	0.13	−0.05	−0.04	0.45	0.38	0.47						
[9] Quiz views	32.0	18.0	0.10	0.04	−0.09	−0.03	0.45	0.88	0.51	0.39					
[10] Lesson clicks	54.9	31.9	0.09	−0.01	−0.06	−0.01	0.58	0.95	0.56	0.45	0.87				
[11] Additional information clicks	14.7	12.5	0.20	0.05	−0.01	0.04	0.52	0.65	0.80	0.66	0.63	0.72			
[12] Assessment clicks	13.4	10.3	0.17	0.07	−0.04	0.05	0.43	0.62	0.49	0.46	0.75	0.71	0.60		
[13] Quiz clicks	25.7	14.3	0.08	0.03	−0.10	−0.05	0.42	0.87	0.46	0.34	0.97	0.83	0.58	0.57	
Learning gains	0.00	0.11	0.07	0.07	−0.10	−0.11	0.25	0.21	0.18	0.32	0.18	0.21	0.12	0.15	0.17

Note. All |*r*| > 0.14 statistically significant at *p* < 0.05, |*r*| > 0.19: *p* < 0.01.

## Data Availability

We provide the online course used as an open educational resource and provide all the data and code underlying this research in an open repository (https://osf.io/gp6h3/).
